# Mr. Paget’s Report Continued

**Published:** 1844-03-23

**Authors:** 


### RECORD OF MEDICAL SCIENCE.

### MR. PAGET'S REPORT CONTINUED.

#### DIGESTION.

*Structure of the teeth.* Mr. Lintott has pointed out the fact of a regular and constant formation of a layer of bone or, probably, of imperfect ivory like what Mr. Nasmyth has called ossified pulp, within the pulp-cavity of the human tooth, after the age of twenty years, independently of any wearing down of the enamel. The layer is thickest at the orifice of the dental cavity, and gradually diminishes as it descends into it till it is lost upon the walls; its thickness increases with advancing age. He remarks also that the part which is by far the most frequent seat of the commencement of decay in the molar teeth is the groove which separates the tubercles of their crowns, and at which the operculæ (according to Mr. Goodsir) meet when the papillary is changed into the capsular stage of development. These grooves are first affected as regularly in the upper as in the lower jaw; as if they were from the first imperfectly developed: [that is, probably, they are liable to the imperfections of parts last formed, such as are often seen in the other *lines of median* or *central fusion.*]

*Salivary secretion.* Dr. Budge has found that after extirpation of the parotid, submaxillary, and sublingual glands in a dog and a rabbit, the secretion of saliva continued; its characters remained the same, and no function was disturbed. [The experiments add probability to the opinion that the labial, buccal, palatine, and other glands which the experimenter left behind, are salivary glands.

A case of a kind of metastasis of the salivary secretion is related by Dr. Roelants, and is interesting in its relation to the general physiology of secretion. A man, eighty-two years old, had an attack of bronchitis, with fever, followed by suppuration around and probably in one of the parotid glands. The abscess was opened, and two months after a large mass of chalk-like substance was discharged. The abscess soon healed, and he recovered his health; but now, whenever he masticates, saliva flows freely from the skin of the cheek and temple of the side formerly diseased. As soon as he begins to eat, the skin becomes very full of blood, and hot; and gradually drop after drop of clear fluid, with all the characters of saliva, collects on its surface, and runs down the cheek and neck, and continues to do so just as long as he continues eating. His health is not disturbed, and the saliva-secreting surface of the skin is natural in its texture.

*Anatomy of the pharynx.* Professor Mayer of Bonn described some time ago a *bursa pharyngea* in many mammalia. He has since found it several times in men. It lies in a corresponding position to that which it occupies in the mammalia, namely, in the middle line in the mucous membrane covering the body of the sphenoid bone, just behind the posterior border of the vomer. It is sometimes large enough to hold a cherry-stone, and in one case was double. He thinks it probable that in other mammalia, in which the bursa is larger, it may sometimes communicate with the sphenoidal sinuses.

*Functions of the stomach.* MM. Sandras and Bouchardat, assuming that, in general, dissolved substances are absorbed by the veins of the stomach, while those that are insoluble are taken into the lacteals, believe that they have proved that the chief classes of aliments are thus disposed of: 1. Fibrin, albumen, caseum, gluten, and the gelatinous tissues are dissolved by the aid of hydrochloric acid; [and, probably, of pepsin.] A mixture of six parts of this acid with 10,000 of water they found sufficient to make all these principles swell up into translucent masses, and *sometimes* to dissolve them. 2. The starchy and saccharine principles are converted wholly or in part into lactic acid, and in that form are absorbed in the stomach. 3. The fatty matters are insoluble, and pass into the intestines, where they are taken up by the lacteals, and form the greater part of the chyle. The experiments which were performed to confirm these opinions before the reporters to the Institute did not succeed well; but if they had done so it would still be hard to explain how the albumen and fibrin can be formed in the chyle from fatty matter alone. Still that some of the starch of food may be transformed and absorbed in the stomach is confirmed by the experiments of Dr. Percy. These make it probable, 1, that sugar is formed in the stomach by the digestion of starch or wheat flour, though neither these experiments, nor any others yet performed, can afford demonstrative evidence of it; 2, that the dextrin into which the starch is first transformed may be at once absorbed, so as to reduce the quantity of sugar which is formed; and 3, that the sugar which is formed must be quickly further changed or absorbed. The latter is the more probable conclusion, and best accounts for the very small quantity of sugar which is ever found after feeding on starch. Lastly, Dr. Percy suggests, that in the cases in which Dr. McGregor found sugar in the stomachs of those diabetic patients who for several days had had only animal food, it might be formed by the oxydation of the fat which is constantly being absorbed from the body during emaciation.

*Composition of the bile.* Dr. Kemp, by careful elementary analysis of the bile of the ox, has corroborated Demarcay's opinion that it is essentially a true chemical compound of an electro-negative body with soda. But he holds that this body is neither the *choleic acid* of Demarcay, since it is not precipitated from the soda by acetic acid, nor the *bilin* of Berzelius, because it is not precipitated from the soda by carbonic acid. He has therefore given it the name of *bilic acid.* It has a peculiar bitter-sweet taste, and in mass resembles a fine resin. It is soluble in every proportion in water. In a subsequent paper he has shown that a much greater difference than is usually imagined is effected in the bile while in the gall-bladder. Bile from the hepatic ducts of an ox was destitute of the bitter taste of cystic bile; its smell also was different. It chiefly consisted of two different electro-negative bodies, separable by alcohol, and each combined with soda.

### ABSORPTION.

M. Lacauchie describes the intestinal villi as possessing during life a power of alternately retracting and elongating themselves by a kind of vermicular motion, which he believes to be influential in the propulsion of chyle. And his account, so far as these movements are concerned, is confirmed by MM. Gruby and Delafond, who have observed them in the recently-slain horse, dog, and rabbit. They add that besides the movements of retraction and elongation, the villa are capable of moving laterally in all directions, and that their epithelium-cells bear ciliæ.

Some experiments by Dr. Behr may serve, perhaps, to explain somewhat of that which was supposed to depend on an elective power of absorption possessed by the lymphatics, and certainly have added much to the probability that the force by which the lymph is carried along the lymphatics is that of the contraction of their walls. It has been long known that the lymphatics will not convey certain substances, especially narcotic poisons, while they do carry others. If, for example, the animal's abdominal aorta be tied so as to stop the circulation in its posterior extremities, and ferrocyanate of potass be inserted in a wound in one of them, it is absorbed and carried into the blood by the lymphatics, and is found again in the urine. But if, under the same circumstances, a narcotic poison is put in the wound the animal is not killed by it; and it was supposed that the lymphatics in this exercised some kind of choice. The results of Dr. Behr's experiments are these: 1. Acetate of strychnine was introduced into a wound in an animal's leg, while the circulation was uninterrupted, and death, with convulsions, &c., occurred in five minutes. 2. Ferrocyanate of potass was introduced into a similar wound, and ten minutes after acetate of strychnine into another wound: in four minutes the animal died of the poison, and the ferrocyanate was found in the urine. 3. The same substances were introduced together into a wound in the leg: the animal died poisoned, and even sooner than before, and the salt was found in the urine. 4. The abdominal aorta was tied below the renal arteries and when the hind limbs were paralysed, the acetate of strychnine was put into a wound in one leg and the ferrocyanate of potass into a wound in the other. After two hours and a half there were no signs of poisoning, but on killing the animal the salt was found in the urine. 5. The abdominal aorta was tied as in No. 4, and the acetate of strychnine and ferrocyanate of potass were introduced into the same wound. The animal showed no signs of poisoning, and the salt could not be found in the urine. This last experiment was several times repeated, and, with unimportant variations, with a constantly similar result. It would follow, therefore, that when the circulation in the blood-vessels is stopped, the lymphatics can absorb and convey to the blood ferrocyanate of potass, but not acetate of strychnine; and that when the two substances are applied to them together it can absorb or carry neither. Hence it is supposed that the force by which the lymphatics convey fluids is that of the contraction of their walls, and that they are paralysed by the direct contact of narcotics, as other involuntary muscles are.

Mr. George Robinson has related some experiments in evidence that the absorption of blood-vessels depends on a force generated by and proportioned to the velocity of the blood which is moving in them. He compares it to that force with which water or any other fluid traversing a main tube will draw fluid through a side branch, even against the weight of a considerable column. He has often repeated this well-known experiment, and has added proof that the same force will act in the same way through one or more membranes. Having filled a wine-glass with coloured fluid, and having connected its contents, (by means of a bent tube twelve inches long and 1-14th of an inch in diameter, and having one of its ends covered with membrane,) with the interior of a pipe half an inch in diameter, he found that within five minutes after the stream had begun to flow rapidly through the last-mentioned pipe, the whole of the air present in the smaller tube was absorbed, and its place supplied by the coloured fluid, which had risen from the glass. In another experiment the fluid from the glass was raised through a shorter tube to the membrane, and was made to flow in a slow but constant stream towards the fluid, passing through the larger pipe.

